# Participatory Research With Older Workers in a Pandemic: Innovations and Lessons Learned

**DOI:** 10.1093/geroni/igab046.985

**Published:** 2021-12-17

**Authors:** Cal Halvorsen, Kelsey Werner, Elizabeth McColloch

**Affiliations:** Boston College, Chestnut Hill, Massachusetts, United States

## Abstract

In the spring of 2020, and as the implications of the COVID-19 pandemic became increasingly dire, in-person studies halted throughout the world. This included our planned study to examine the role of the Senior Community Service Employment Program (SCSEP)—the sole federal workforce training program for low-income older adults—in influencing participant financial, physical, and mental well-being. While our original plans were to hold a series of in-person workshops with SCSEP participants and case managers using a form of participatory research called community-based system dynamics (CBSD), we paused the launch of our study to determine the safest path forward. This presentation will describe how we responded as well as innovations and implications for future research with harder to reach populations. First, we met with the Massachusetts state SCSEP director to assess the feasibility of moving our sessions online with this particular population. After determining that virtual and telephone sessions would both be needed to increase accessibility, we identified virtual whiteboard software rigorous enough to utilize CBSD-specific activities, user-friendly enough for populations less familiar with virtual environments, and with security features that would be approved by our university, as well as discussed what types of activities to conduct on the telephone for such a visual research method. Our CBSD study was one of the first to utilize virtual and telephone formats in the history of this method, and our results indicate that it is possible—and sometimes beneficial—to move in-person participatory methods to these environments to increase inclusion and efficiency.

